# A case of diffuse cavernous hemangioma of the appendix: laparoscopic surgery can facilitate diagnosis and treatment

**DOI:** 10.1186/s40792-016-0276-9

**Published:** 2017-01-04

**Authors:** Chisato Takagi, Kazuo Yamafuji, Hidena Takahashi, Atsunori Asami, Kaoru Takeshima, Hideo Baba, Nobuhiko Okamoto, Kiyoshi Kubochi

**Affiliations:** Department of Surgery, Saitama City Hospital, 2460 Mimuro, Midori-ku, Saitama City, Saitama 336-8522 Japan

**Keywords:** Vascular malformation, Cavernous hemangioma, Chronic abdominal pain, Appendicitis, Laparoscopic surgery

## Abstract

**Background:**

A cavenous hemangioma of the appendix (CHA) is rare. The clinical pathophysiology and adequate management of a CHA have not been sufficiently explained since reports on CHA are scarce.

**Case presentation:**

A 56-year-old woman presented with chronic right lower quadrant pain. Abdominal contrast-enhanced computed tomography revealed a thickened appendix (1.5 cm in diameter) and some focal calcifications in the appendiceal wall. No acute inflammatory signs were visible around the appendix. For diagnosis and treatment, we performed a laparoscopic surgery. Intraoperative findings included purple granular lesions that were spread diffusely along the surface of the appendix. Since these lesions were spread to the terminal ileum, laparoscopic ileocecal resection was performed. Upon macroscopic inspection, purple-colored, raspberry-like lesions were found diffusely on the serosal surface of the appendix. No lesions were found on the mucosal surface. Hematoxylin and eosin staining indicated the presence of blood-filled sinus-like spaces largely in the subserosal layer. Immunohistochemistry analysis indicated that CD34-positive cells lined these spaces. Given these findings, we diagnosed the patient with a diffuse cavernous vascular malformation of the appendix.

**Conclusions:**

CHA is difficult to diagnose. A laparoscopic approach may be useful for both the diagnosis and treatment of the disease.

## Background

Hemangiomas of the gastrointestinal tract are infrequently encountered. They are also referred to as vascular malformations since their pathology consists of a collection of malformed blood vessels [1]. Although most cases are asymptomatic, they can present with overt or occult bleeding in small and large intestinal lesions [2, 3].

Cavernous hemangioma of the appendix (CHA) is rare, and reports of CHA are limited and have been written in English, Russian, Japanese, German, and French [3–7]. Thus, their clinical pathophysiology and adequate management have not been explained sufficiently and are not readily available to an international audience.

In this article, we report a case of diffuse CHA, which was resected using a laparoscopic ileocecal procedure.

## Case presentation

**Fig. 1:**
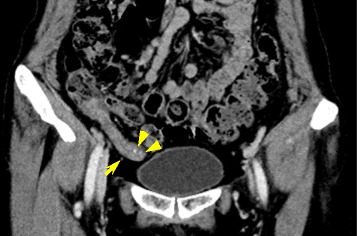
Contrast-enhanced abdominal computed tomography (CT). A thickened appendix, without signs of inflammation, including fat stranding and extra-luminal fluid, was observed in the coronal plane (*yellow arrow*). There were focal calcifications in the appendiceal wall (*yellow arrowheads*)

A 56-year-old woman presented to our hospital with right lower quadrant pain. She experienced pain once every few months beginning a few years previously. Her abdomen was soft, with no peritoneal irritation signs or palpable masses. An abdominal contrast-enhanced computed tomography (CT) revealed a thickened appendix (1.5 cm in diameter) and some focal calcifications in the appendiceal wall. Abnormal findings were not seen in the terminal ileum. There was no evidence of tumor and no acute inflammatory signs (Fig. 1). Colonoscopy showed no mucosal lesions in the appendiceal orifice and cecum. Owing to the lack of findings requiring emergency treatment, we followed her without therapy for 2 months. After 2 months, we performed an abdominal contrast-enhanced CT again because her abdominal pain did not alleviate. The findings in the appendix were the same as those obtained earlier. We then assumed that the patient had chronic appendicitis and performed a laparoscopic surgery to diagnose and treat the condition.

**Fig. 2:**
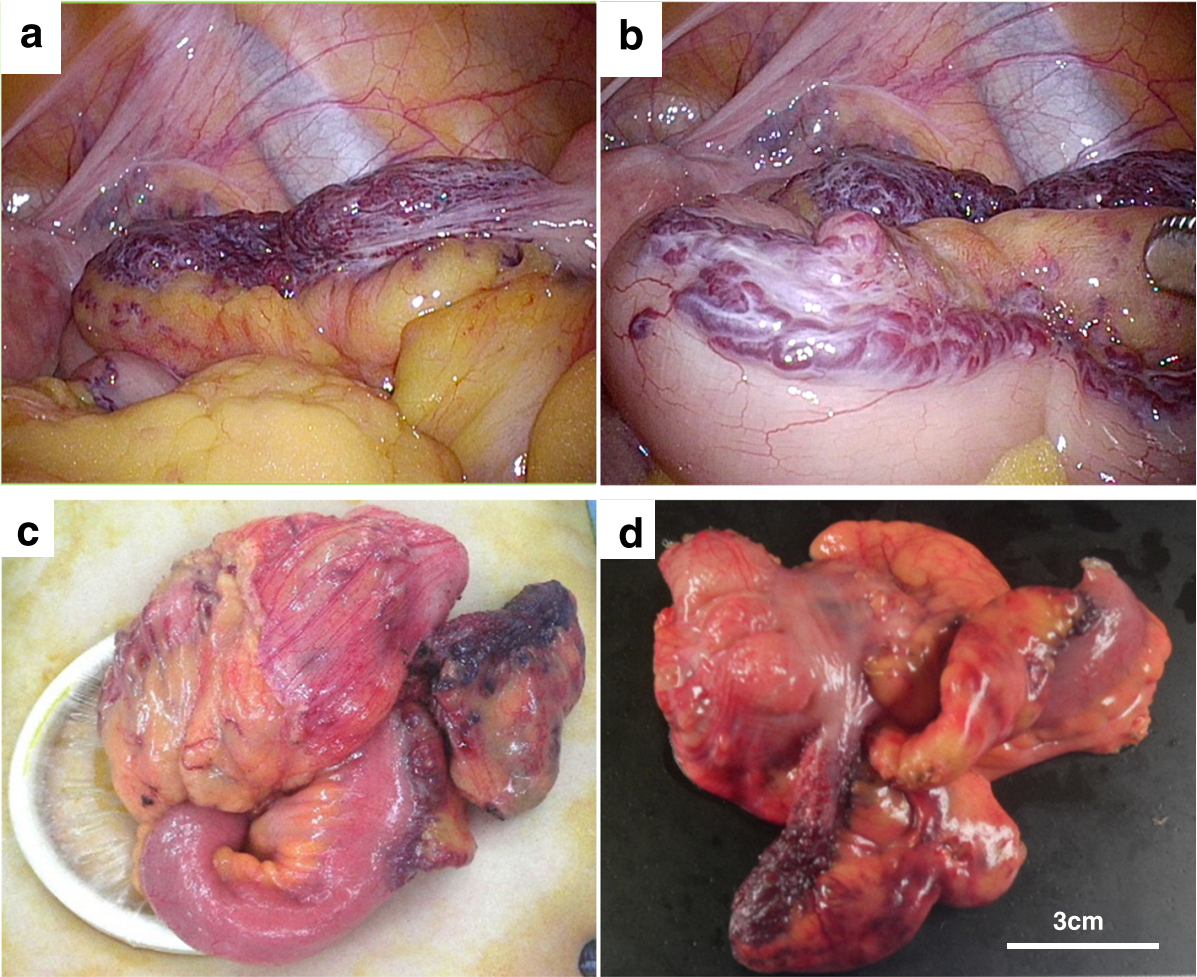
Intraoperative findings and outer image of the resected ileocecum. **a** Purple-colored nodular or granular lesions were presented with a raspberry-like appearance that spread diffusely along the serosal surface of the appendix. **b** The lesion-like appendix spread to the terminal ileum. **c** The ileocecum was mobilized and elevated outside the body before resection. The entire lesion is observed. **d** The resected ileocecum contains the entire lesion

Intraoperative findings included purple-colored, granular lesions that were raspberry-like in appearance and were spread diffusely along the surface of the appendix (Fig. 2a). These lesions spread to the terminal ileum (Fig. 2b, c). Therefore, laparoscopic ileocecal resection was performed to completely resect the lesion. The resected ileocecum contained approximately 6 cm of the ileum (Fig. 2d). She was discharged with an uneventful postoperative course. After surgery, her abdominal pain alleviated.

**Fig. 3:**
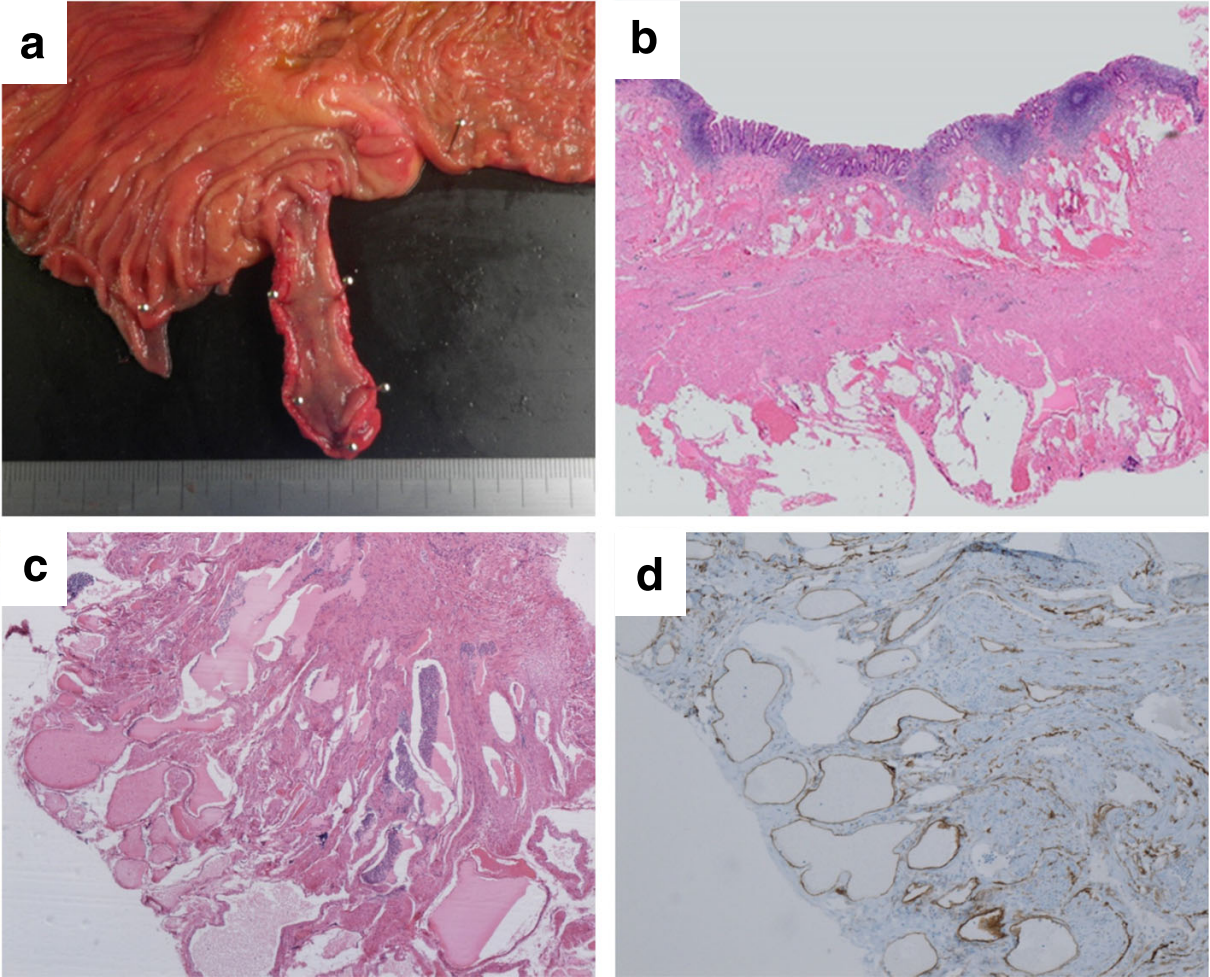
Macroscopic appearance and histological examination of the resected specimen. **a** No lesions were found on the mucosal surface. **b** There were sinus-like spaces in the submucosal, proper muscular, and subserosal layers of the appendix, as indicated by hematoxylin and eosin staining. **c** Some of these spaces were filled with blood. **d** CD34-positive cells were lined with vascular spaces on immunohistochemistry analysis

Macroscopic inspection of the resected specimen revealed purple-colored, raspberry-like lesions, which were spread diffusely on the serosal surface of the appendix. No lesions were found on the mucosal surface (Fig. 3a). Histological examination indicated that there were large and small sinus-like spaces, largely in the subserosal layer. The same finding was also observed in the submucosal and proper muscular layer (Fig. 3b). Some of these spaces were filled with blood (Fig. 3c). Cells that lined the vascular spaces were strongly immunoreactive for CD34, which is one of the main markers of endothelial cells (Fig. 3d). These sinus-like spaces were thought to be malformed vessels. Although there was chronic inflammation in the mucosal layer of the appendix, the structure of the mucosal layer was not injured. Finally, we diagnosed the patient with diffuse cavernous vascular malformation of the appendix.

### Discussion

As shown in this case, unexplained chronic right lower quadrant pain and appendiceal wall thickening with focal calcification, as evidenced by CT, may possibly be a case of CHA, which is otherwise difficult to diagnose preoperatively. Owing to limited available reports, clinical manifestations and findings of various diagnostic images of CHA are not well-known. Previously, CHA was reported to cause no symptoms. For this reason, it was only incidentally discovered during a surgery to treat other lesions [3, 5]. Another report indicated that intraperitoneal bleeding was caused by rupture of the CHA, which was also not diagnosed preoperatively [6]. Another case was reported along with acute appendicitis, which was diagnosed preoperatively [7]. Previous reports involving diagnostic imaging demonstrated that CT, magnetic resonance imaging (MRI), and colonoscopy were unable to detect the lesion [5]. Meanwhile, in other reports, no diagnostic imaging modalities were employed and CHA was not diagnosed preoperatively [3, 6]. Gastrointestinal cavernous hemangiomas can cause pain and gastrointestinal bleeding [8, 9]. Furthermore, CT imaging can detect focal calcification in gastrointestinal hemangioma as a degenerative change [10]. Gastrointestinal endoscopy, including esophagogastroduodenoscopy, colonoscopy, and capsule endoscopy, can also be useful diagnostic tools for gastrointestinal cavernous hemangioma [8]. While the chronic right lower quadrant pain and the appendiceal wall thickening with focal calcifications that were observed by CT in this study have not been described for CHA previously, they corroborate the pathology of CHA. It is possible that obstruction of the appendiceal lumen and congestion of the intestinal fluid in the appendix can cause abdominal pain. Since a colonoscopy can reveal only the appendiceal orifice, this examination might not fully contribute to a diagnosis of CHA. However, a colonoscopy may also be necessary to rule out other possible diseases such as cancer or inflammatory diseases.

This case also demonstrates that laparoscopic surgery can be a useful tool for both diagnosis and treatment. Diagnostic laparoscopy for suspected appendicitis has been shown to be useful for its diagnosis and treatment because it is usually a simple and safe procedure that is helpful in obtaining other diagnoses [11]. CHA has specific findings, such as a purple-colored nodular or granular lesion that is raspberry-like in appearance, which can be seen only during surgery [3, 5]. In the present case, the same finding was observed and laparoscopic resection was performed. A conservative surgical resection, such as laparoscopic surgery, was thought to be a sufficient treatment in order to relieve symptoms or prevent complications caused by CHA. To the best of our knowledge, this is the first report wherein CHA was observed and resected using a laparoscopic approach.

Similarly, cavernous vascular malformation of the mesoappendix has been reported [9, 12, 13]. Like CHA, cavernous vascular malformation of the mesoappendix is a rare disease and can form a tumor that results in abdominal pain. CT and MRI can provide informative findings for diagnosis. The relationship between appendiceal cavernous hemangioma and mesoappendicial cavernous hemangioma are not well known. To better understand the pathophysiology, further clinical data are required.

## Conclusions

CHA is a rare disease that is difficult to diagnose by history taking, physical examinations, and various diagnostic imaging modalities because of its rarity. However, unexplained chronic right lower quadrant pain and appendiceal wall thickening with focal calcification, as indicated by CT imaging, may provide a basis for suspecting a diagnosis of CHA. A laparoscopic approach may be useful for both the diagnosis and treatment of CHA.

## Abbreviations

CHA: Cavenous hemangioma of the appendix
CT: Computed tomography
MRI: Magnetic resonance imaging
